# HeLa-CCL2 cell heterogeneity studied by single-cell DNA and RNA sequencing

**DOI:** 10.1371/journal.pone.0225466

**Published:** 2019-12-02

**Authors:** Wan-er Hu, Xin Zhang, Qiu-fang Guo, Jing-wei Yang, Yuan Yang, Shi-cheng Wei, Xiao-dong Su

**Affiliations:** 1 Academy for Advanced Interdisciplinary Studies (AAIS), Peking University, Beijing, China; 2 Biomedical Pioneering Innovation Center (BIOPIC), and State Key Laboratory of Protein and Plant Gene Research, Peking University, Beijing, China; 3 Clinical Research Center, Guizhou Medical University Hospital, Guiyang, China; Indraprastha Institute of Information Technology Delhi, INDIA

## Abstract

The HeLa cells are the earliest and mostly used laboratory human cells for biomedical particularly cancer research. They were derived from a patient’s cervical cancerous tissue, and are known for their heterogeneous cellular origin and variable genomic landscapes. Single-cell sequencing techniques with faithful linear and uniformly amplified genomes (DNA) and transcriptomes (RNA) may facilitate the study of cellular differences at the individual cell level. In this work, we have performed single-cell DNA and RNA sequencing with HeLa-CCL2 cells to study their heterogeneity. We have studied the complexity of copy number variations (CNVs) of HeLa-CCL2 genome at the single cell level, and revealed the transcriptomic heterogeneity of HeLa-CCL2. We also analyzed the relationship between genome and transcriptome at the single-cell level, and found overall correlation between CNV and transcriptome expression patterns. Finally, we concluded that although single-cell sequencing techniques are applicable to study heterogeneous cells such as HeLa-CCL2, the data analyses need to be more careful and well controlled.

## Introduction

In 1951, Gey *et al*. isolated the cancer tissue from Henrietta Lacks, a woman with cervical cancer, and established the HeLa cell line *in vitro*, which was the first human-derived immortalized cell line [1]. In the next sixty plus years, HeLa became the most wildly used cell line in biomedical research, and produced results for more than 70,000 publications (searched from PubMed).

Since the establishment, the HeLa cells had undergone multiple generations of propagations. HeLa cells have derived more than 300 progeny strains utilized in many fields of life sciences. The HeLa-S3 cell line was separated from HeLa-CCL2 in 1955 [2], and the HeLa-Kyoto was isolated in 1980s by Narumiya of Kyoto University, Japan. HeLa-S3 can grow as suspension cultures, and is widely used in the field of cytology. The morphology of HeLa-Kyoto is more favorable for imaging, and there are massive progeny cell lines derived from it that are transfected with different fluorescent proteins. However, the most widely used and distributed HeLa cells are HeLa-CCL2 which is considered to be the direct progeny of the original HeLa cells. Although it has long been observed by cell biologists that HeLa cells are quite abnormal and heterogeneous, containing high aneuploidy and variable chromosomal organizations, the whole genome landscapes of HeLa cells were only described recently after NGS (next-generation sequencing) became prevailing in 2013 [3, 4]. These studies have established accurately the aneuploidy and genomic heterogeneity of the HeLa cells and shown that HeLa was in average hypertriploid (3n+), which is consistent with early G-Banding karyotype results [5]. These works revealed complex genomic landscapes of HeLa that displayed up to 30+ chromosome-level large fragment translocations, including over 2000+ fragments (>50 bp) translocations [3]. To understand the genomic and transcriptomic variability caused by culture conditions, Frattini *et al*. [6] compared 4 different HeLa-CCL2 strains that were cultured in 4 different laboratories. Alarmingly, the results suggested that the same experiment performed on the HeLa cells cultured in different labs could lead to distinct conclusions and irreproducible results. Similar observations were made in some breast cancer cell lines [7].

Tumor heterogeneity refers to the fact that different cancer cells display signifiant differences in phenotype, methylation status, transcriptome, and particularly genome. Intratumor heterogeneity results from chromosomal instability [8–10], which can lead to somatic mutations, ranging from single nucleotide variations (SNVs) to chromosome structure changes, such as copy number variations (CNVs) and even to larger changes at the entire chromosome level. Intratumoral heterogeneity consists of both spatial heterogeneity and temporal heterogeneity. In the study of the primary kidney tumor, Gerlinger *et al*. [9] constructed a phylogenetic tree of tumors and pointed out that targeted treatment based on primary tumor biopsy results may not be effective in treating metastases. All in all, tumors display a complex dynamic ecosystem. The internal tumor heterogeneity, consisting of a large number of mutations and chromosomal changes, initiates and promotes cancer evolutionary processes.

Conventional high-throughput (HTP) sequencing already has a tremendous impact on the field of biomedical research. In this method, it is necessary to obtain enough DNA or RNA from a large number (typically millions) of cells for sequencing, and the sequencing results are the averaged representation of all these cells. However, due to cellular heterogeneity, particularly for cancer cells, there might be significant genetic differences in these cells. Moreover, low-abundance changes would be easily lost in the overall averaged characterization. As for the whole genome single-cell DNA sequencing (scDNA-seq), the priority and difficulty was to develop the whole genome amplification (WGA) technology owing to the fact that the amount of intracellular DNA is extremely small (only one copy of DNA for haploids and two copies for diploids; a human diploid cell typically contains 6 pg of genomic DNA). The WGA technology has undergone quite a few technological changes: first appeared in chronological order was a WGA method based on polymerase chain reaction (PCR) in 1992, such as primer extension pre-amplification PCR (PEP-PCR) [11] and degenerate oligonucleotide primed PCR, (DOP-PCR) [12]; but PCR-based methods suffer from enormous exponentially amplified regions that can cause non-linear amplification bias. WGA based on constant temperature reaction, such as multiple displacement amplification (MDA) could largely overcome non-linear amplification bias [13]. Later in 2012, the other development with WGA was multiple annealing and looping-based amplification Cycles (MALBAC) [14]. He *et al*. [15] assessed the performance of MDA and MALBAC for β-thalassemia genotyping and single-nucleotide polymorphism (SNP) / CNVs detection. When performing CNVs detection at the single cell level, they found that MALBAC has better stability than MDA. To compensate for the limitations of conventional NGS sequencing, single-cell RNA sequencing (scRNA-seq) technology has been developed during the past 10 years [16]. Faithful single-cell sequencing could reveal the degree of variation among individual cells. Tang *et al*. has taken the lead on the development of HTP scRNA-seq in 2009 [16] based on previous single-cell microarray techniques [17, 18]. In 2012, SMART-Seq (Switching mechanism at 5’ end of the RNA transcript sequencing) [19] came out and became a robust method for full-length mRNA sequencing. An updated version of this technique, SMART-Seq2, was published in 2014 [20]. Later, Tang [21] modified SMART-Seq2 with the introduction of a specific reverse transcription primer, which was a 25 nt oligo (dT) primer anchored with an 8 nt (nucleotide) cell-specific barcode and 8 nt unique molecular identifiers (UMIs) [22–25]. This protocol detected the 3’end of mRNA and made it more high-throughput, accurate and cost effective.

Current researches on the extremely heterogeneous HeLa cells have set the landscapes of HeLa cell karyotyping, genomic and transcriptomic diversities on the bulk sequencing level. Studies on HTP whole genome sequencing of HeLa cell lines at single-cell level have never been published to our knowledge. In this work, we performed scDNA-seq and scRNA-seq on 20 and 720 HeLa-CCL2 cells, respectively. We aimed to test the idea if we can construct a heteroploidy map by CNVs detection performed by scDNA-seq and build subtype classification of HeLa-CCL2 cells by different gene expression through scRNA-seq.

## Materials and methods

### 2.1 Ethic statement

The study was approved by the Ethical Committee on Human Research of Peking University and was in accordance with the Declaration of Helsinki. The blood was obtained with informed consent, which confirmed that the donor voluntarily donated blood.

### 2.2 Cell line source and identification

We purchased the cell line HeLa-CCL2 from ATCC (American type culture collection). Cell line identification was performed by Shanghai Biowing Applied Biotechnology Co. Ltd. Short tandem repeat (STR) was applied to identify the cell line, by comparing it to 16 STR profiles from DSMZ (Deutsche Sammlung von Mikroorganismen und Zellkulturen) database including seven markers ATCC set as standard reference profiles for HeLa-CCL2. Other 2 glioma-drived cell lines (U87, U251) were obtained from Prof. Fan’s lab of Beijing Normal University (originally also from ATCC).

### 2.3 Cell culture

HeLa-CCL2 cells were cultured for 9 passages, 14 passages and 20 passages in the medium containing 4.5 g/L glucose (Dulbecco’s Modified Eagle Medium, Gibco), 5% heat-inactivated fetal bovine serum (Vistech) and 1% penicillin-streptomycin solution (10,000 U/ml Penicillin, 10,000 ug/ml Streptomycin, HyClone) and incubate at 37°C and 5% CO2. Cells were harvest at 90% confluence by 2.5% trypsin-EDTA (1X, Gibco) and incubated at 37°C for 2 minutes. For this study, we picked the cells for scRNA-seq at the 9^th^, 14^th^ and 20^th^ passages.

### 2.4 Karyotyping

Karyotype was performed by HyperCyte Biomedical Co. We picked out 150 cells in metaphase and counted the chromosome number under the microscope Olympus CX40.

### 2.5 Single-cell DNA sequencing

We tested the whole genome of 20 HeLa-CCL2 cells. As control, 10 normal human leukocytes from the blood of a healthy volunteer were also sequenced. Add 3 volumes of red blood cell lysis buffer (RBC Lysis Buffer, CWBiotech) to the blood sample and put the sample on ice for 15 minutes. Then centrifuged the sample at 1200 rpm for 5 minutes to removed supernatant and add 1.0 ml DPBS (1X Dulbecco’s Phosphate Buffered Saline, without calcium and magnesium, Corning) to resuspend the leukocytes. Single cells were captured by mouth pipette under the stereomicroscope (Nikon SZM745). The single-cell DNA amplification was followed by the MALBAC technique developed by Xie’s laboratory [14]. After amplification, six genomic loci were checked in the samples by qPCR before generating library to make sure the DNA was well presented as quality control (QC). We picked the sample with over two detectable loci to generate library. The sequencing depth for a single cell was 0.3X (about 1G per cell).

### 2.6 HeLa-CCL2 whole genome DNA sequencing

We harvested 1.0 x 10^7 HeLa-CCL2 cells and extract the whole genome DNA with QIAGEN Blood & Cell Culture DNA Midi Kit (Cat.No.13343). The samples were fragmented to 300 bp by S220 Focused-ultrasonicators (Covaris, USA). And DNA library was generated by NEBNext Ultra DNA Prep Kit for Illumina (E7370L). The sequencing depth for bulk DNA was 1X (3G).

### 2.7 Single-cell RNA sequencing

We randomly chose 720 HeLa-CCL2 cells (288 from the 9^th^ passage, 288 from the 14^th^ passage and 144 from the 20^th^ passage) in this work on an Illumina HiSeq 4000 platform (sequenced by Novogene, China) for 150 bp paired-end sequencing. The protocol was totally followed Tang’s modification on SMART-Seq2 [21]. Single cells were captured by mouth pipette under the stereomicroscope (Nikon SZM745) and followed by the protocol [21]. Every cell was assigned with 250 M raw data on average.

### 2.8 Single-cell DNA sequencing and bulk DNA sequencing analysis

The aligned to the hg19 human genome sequence (UCSC) using BWA [26]. To exclude the pseudoautosomal regions shared by both chromosome X and chromosome Y, we replaced the pseudoautosomal region sequence with N in the hg19 sequence. After quality control, we mapped the reads into 200 kilobase bins separating the genome, each having the same number of mappable positions. And to analyze CNVs of the cell, we normalized the bin counts with GC content of different bins using LOWESS smoothing and segmented the bins into blocks differing in copy number using CBS segmentor [27].

### 2.9 Single-cell RNA sequencing analysis

Raw reads in a library were separated first by different barcodes into different cells. Then we recorded the reads with different UMIs and trimmed the template switch oligo sequence and poly-A tail sequence [21]. After quality control, the clean reads were mapped to the hg38 human transcriptome (UCSC) using hisat2 [28, 29]. And then we counted the mapped reads with different UMI using HTSeq tools [30]. The reads that had the same UMI were counted once in the final gene expression matrix. With the gene expression matrix obtained above we analyzed the transcriptome expression of the 720 HeLa cells using Seurat3.0 R package [31]. We filtered the cells expressed less than 2000 genes and genes expressed in less than 10 cells. The data was scaled after log normalization. And we performed principal component analysis (PCA) and Uniform Manifold Approximation and Projection (UMAP). The PCA analysis was performed based on highly variable genes. Before PCA, we used Harmony for batch correction among 3 generations [32] within the Seurat3.0 workflow, using the default parameters. Next, InferCNV package was applied to infer CNV from scRNA-seq data [33]. By exploring expression intensity of genes across positions of tumor genome, a heatmap is generated illustrating the relative expression intensities across each chromosome [33]. Gene ontology analysis was performed using clusterProfiler [34], following the GO analysis part of the R package using default parameters.

## Results

### 3.1 The karyotype result confirmed the aneuploidy within HeLa-CCL2

For the verification that HeLa-CCL2 cells are mixture of aneuploidy cells, we applied the nuclear staining experiment. We picked 150 cells during metaphases to count their chromosomes. The result (S1 Fig) showed that almost 85% cells with chromosome number ranged from 63–70, corresponding to the triploid (69) model as suggested in the past [3, 5].

### 3.2 Single-cell DNA sequencing results depicted the heterogeneous characters of HeLa-CCL2 CNVs with low resolution

To investigate the heterogeneity of HeLa-CCL2 genome at the single cell level, we performed MALBAC on 20 HeLa-CCL2 cells. We also carried out MALBAC on 10 leukocytes from a healthy female volunteer and bulk DNA sequencing on HeLa-CCL2 as positive controls. Meanwhile, we compared our MALBAC results with the oocyte cells MALBAC data from Hou [35] with the same analyses method.

#### Intracellular heterogeneity

**Fig 1:**
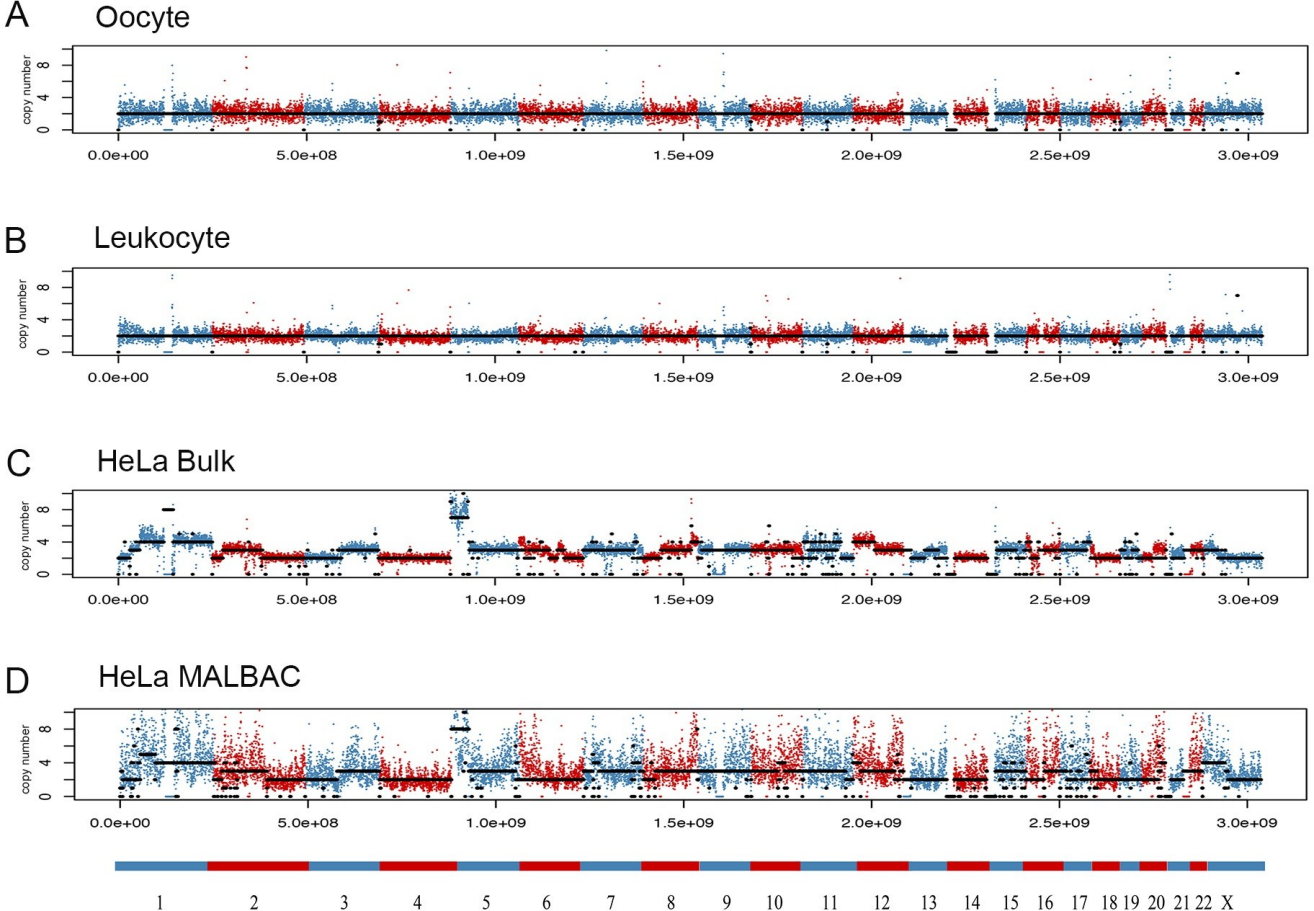
MALBAC and bulk DNA sequencing results. The y axis shows the copy number, and the x axis is the genomic position of hg19. Adjacent chromosomes are marked with different colors. Points are bins with length 200kb. (A) MALBAC results of an oocyte from Hou’s work [35]. (B) MALBAC control results of a leukocyte from a healthy woman. (C) HeLa-CCL2 bulk DNA sequencing. (D) MALBAC results of one typical HeLa-CCL2 cell.

We separated the hg19 genome into bins with length 200 kb and counted the number of reads that mapped to different bins and got the CNV results (Fig 1). According to the variation of bin counts, it showed that the quality of the leukocyte MALBAC data (Fig 1B) was comparative with the data from Hou (Fig 1A), as standard diploid. And the bulk HeLa DNA sequencing showed a complex but clear CNV pattern (Fig 1C), and comparable with previously published data [3, 4]. However, MALBAC data of HeLa cells (Fig 1D) showed so large variation of the bin counts that it was difficult to call precise copy numbers, especially at the region with large CNVs. In general, longer chromosomes showed better amplification uniformity than the shorter ones, especially with those chromosomes close to normal diploid. For example, chromosome 4 were easier to tell the exact copy number and showed similar data quality with normal diploid cells (Fig 1A and 1B). The complex regions with large copy number variations were too noisy to show the actual CNVs clearly. All the control experiments showed that the MALBAC experiments we performed were technically sound, but the quality of data was not satisfactory for further detailed analyses for HeLa. Compared to its bulk DNA sequencing patterns (Fig 1C), which showed complexity and high variability for most chromosomes, we could conclude that the complex intracellular chromosomal heterogeneity led to poor MALBAC data in HeLa.

#### Intercellular heterogeneity

**Fig 2:**
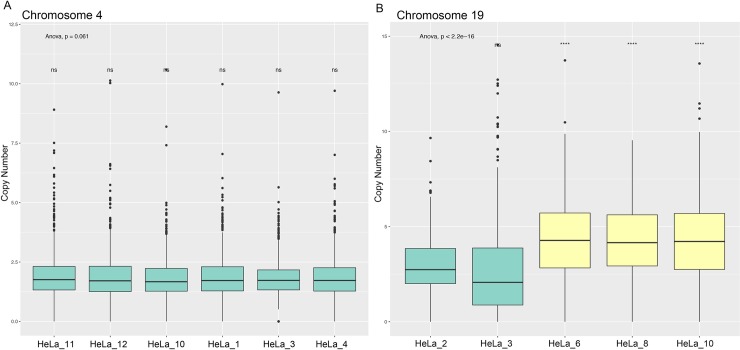
The boxplot of copy number on chromosome 4 and chromosome 19. (A) The boxplot of copy number on chromosome 4 (Anova, P = 0.061). (B) The boxplot of copy number on chromosome 19. The copy number on chromosome 19 showed 2 types. HeLa_6, 8, 10 represented cells with higher copy number (Anova among HeLa_6, 8, 10, P>0.74) and HeLa_2, 3 represented lower copy number (Anova among HeLa_2, 3, P>0.28) on chromosome 19. The statistical difference showed on each box was obtained on the basis of comparison between each cells and HeLa_2.

We next investigated on the genomic CNV differences among random chosen single cells, known as complex triploid model. Despite the low resolution that could only give a rough copy number estimation, we still found different copy number pattern among single cells. Our analysis above suggested that MALBAC didn’t work well on large fragments with complex CNVs. Therefore, we tried to analyze on the chromosome with short length and whose bulk data implied fewer CNVs. We counted the bins of chromosomes with fewer CNVs and compared the significant differences among them. As control, we found no copy number difference on chromosome 4 (Anova, P = 0.061) among cells (Fig 2A). While the copy number on chromosome 19 was divided into two categories. It was obvious that one class of cells had higher copy number while the other had lower copy number (Anova, P<2.2e-16). Although the exact copy number of the two types of cells could not be calculated accurately due to data quality problems, statistical analysis showed that the copy number between the two types of cells was significantly different. The results above clearly indicated the intercellular genomic heterogeneity within HeLa-CCL2 cells.

### 3.3 Single-cell RNA sequencing suggested heterogeneity of HeLa-CCL2 transcriptome

**Fig 3:**
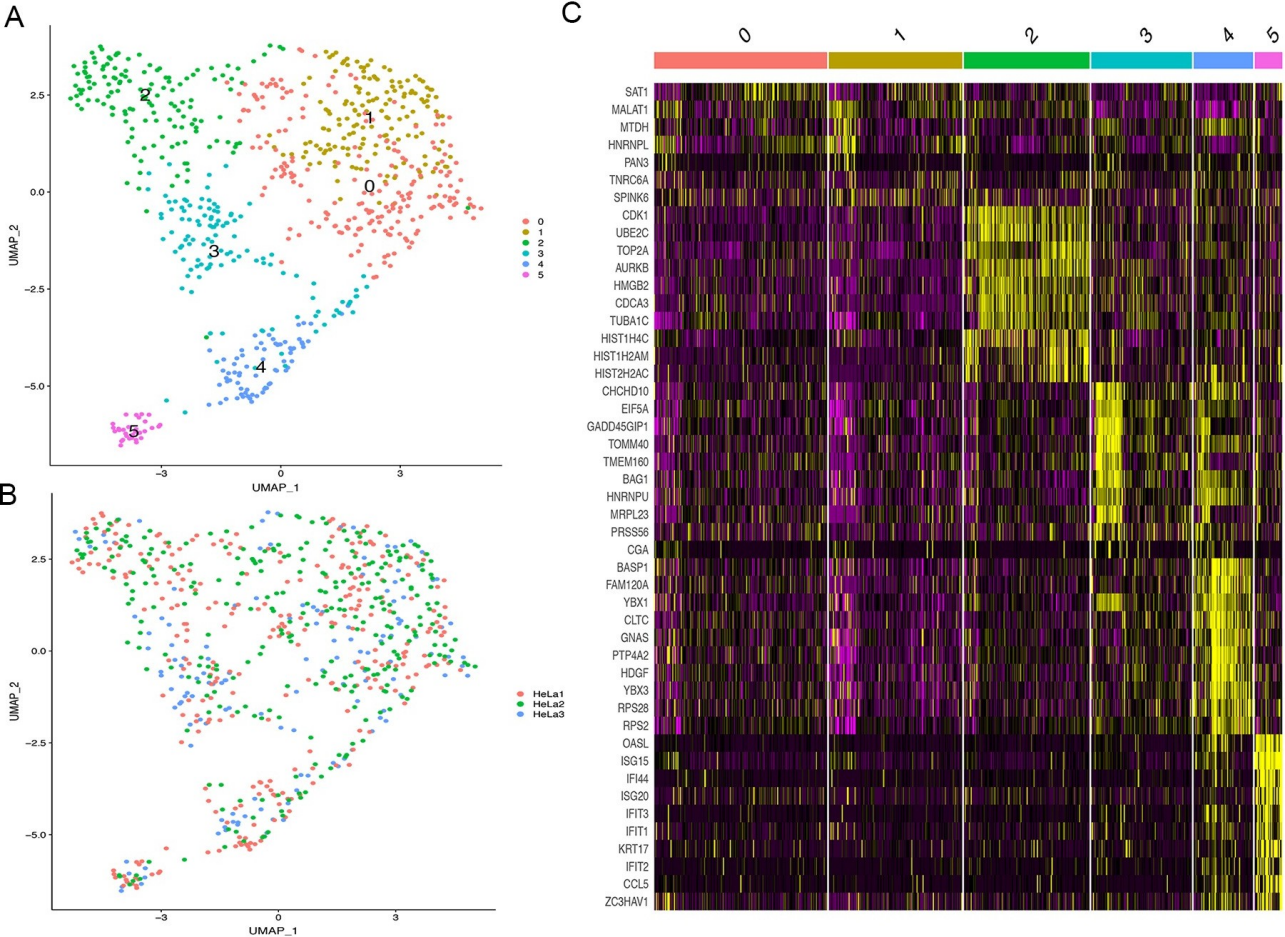
Single-cell RNA sequencing results of 704 HeLa-CCL2 cells. (A) The UMAP plot of 704 cells (every point represented one cell) clarified into 6 clusters for 6 colors. The number of each cluster corresponded to Fig 3C. (B) The same UMAP plot as Fig 3A but the color represented different-passage derived cells. HeLa 1, 2, 3 represented the 9^th^, 14^th^, 20^th^ HeLa-CCL2, respectively. (C) The heat map of the 6 clusters. Yellow stands for high expression and purple for low expression. The genes showing on the right were the top 10 expression genes in every cluster. Cluster 0 and 1 showed overlapped expression with less than 10 genes.

In order to make a comprehensive understanding of the heterogeneity of HeLa-CCL2, we also performed scRNA-seq on 720 HeLa-CCL2 cells from different passages (the 9^th^, 14^th^ and 20^th^). After filtration, 704 cells were processed to further analysis. We acquired about 1.6 million reads and 13504 genes per cell on average. We run a Uniform Manifold Approximation and Projection (UMAP) analysis on 704 filtrated cells and identified 6 clusters (Fig 3A). There were some expression patterns overlapped between cluster 0 and 1, as well as cluster 3 and 4 (Fig 3C). The top 10 expressed genes of cluster 2 were mainly related to cell cycle and mitosis (Fig 3C), suggesting their proliferative characters. While in cluster 5, the top 10 expressed genes were mainly interferon induced protein related (Interferon Induced Protein with Tetratricopeptide Repeats 1, 2, 3 (*IFIT1*, *2*, *3*), Interferon-Stimulated Protein (*ISG15*), Interferon Stimulated Exonuclease Gene 20 (*ISG20*)) (Fig 3C). Highly expressed genes in cluster 3 and 4 were hard to category into a single biological function.

**Fig 4:**
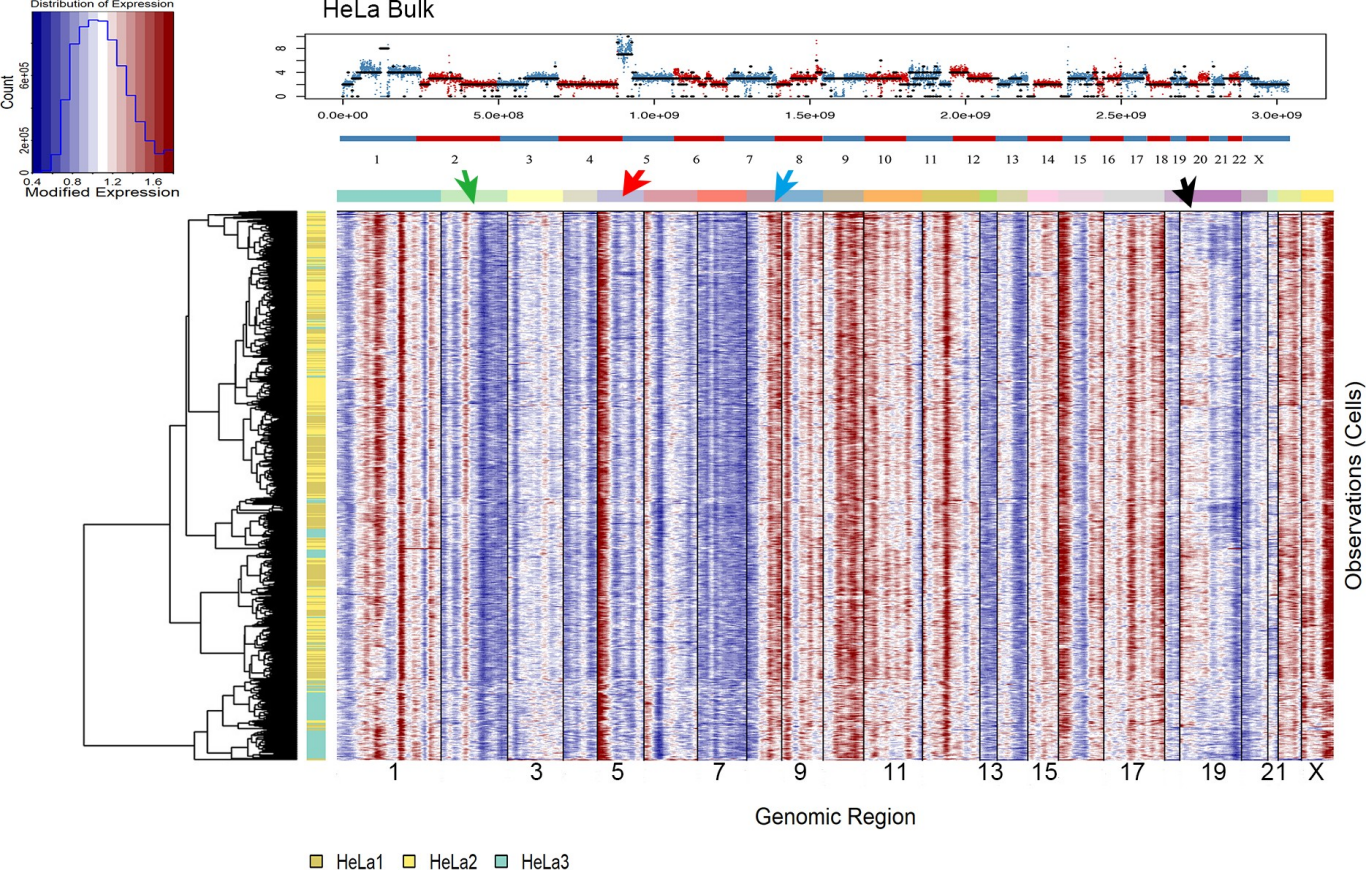
The inferred CNV pattern based on scRNA results. The y axis represents each cell, and the x axis is the genomic position of hg19. CNV on chromosome 19 showed 2 types of CNV patterns. The HeLa-CCL2 bulk DNA sequencing result is placed here to make it easier to compare with the trend between bulk DNA and the inferred CNV patterns. Different arrows are helped to point out the similar trend between HeLa bulk DNA result and the inferred CNV pattern.

Next, we inferred CNVs of the HeLa cells based on the scRNA-seq data (Fig 4). The scRNA-seq data CNV inferring results showed similar CNV pattern with bulk DNA results from a large scale. For example, the copy number on chromosome 2 had an overall three-stage change, and the same trend could be seen in the inferred CNV map (Fig 4 green arrow). Chromosome 5 in bulk DNA showed high copy number and 3 copy number, while in inferred CNV figure it showed the same pattern on chromosome 5 (Fig 4 red arrow). The copy number on chromosome 8 displayed a change from low to high both on bulk and inferred CNV (Fig 4 blue arrow). While looked at chromosome 19, we found that a large proportion of the single cells showed similar copy number with the rest cells showed lower (Fig 4 pointed out by black arrow), corresponding to the 2 types of copy number pattern presented in the MALBAC data (Fig 2). These results indicated that scRNA-seq and bulk/single-cell DNA-seq were mutually complementary.

## Discussion

In this study, we have firstly confirmed the karyotype status of our HeLa-CCL2 cells. We then chose 150 cells in metaphase to count their actual chromosomes. The raw data was shown in S1 Table. The results showed that our HeLa cell aneuploidy was consistent with previous studies and ATCC standard [3].

To get comprehensive understanding on HeLa-CCL2 heterogeneity, we intended to construct the heteroploidy map of individual HeLa cells by CNV detection conducted by MALBAC. We have analyzed 20 HeLa cells but only got rough results because of the low resolution and large noise. We also performed MDA on HeLa cells but got even worse results (S2 Fig). With these controls (Fig 1A and 1B), we ruled out the experimental operation that might lead to the bad resolution. According to the bulk result (Fig 1C), the HeLa-CCL2 cells showed complex CNV patterns all over the whole genome, in which the CNV changed dramatically over a short genomic distance such as chromosome 1, 5, 8, 11, 16. The complexity of the HeLa-CCL2 genome made it difficult to call CNV with single-cell MALBAC data. Otherwise, the single-cell DNA-seq results maintained the same trend overall with bulk DNA-seq. To sum up, we think that MALBAC did not work very well on HeLa cells attributed to the very large intracellular chromosome heterogeneity although it worked well for tumor cells consisting small CNV changes [36].

Even though the copy number analysis results were not optimal we still detected copy number differences at the chromosome level among different cells. The distinct CNV differences on chromosome 19 (Fig 2) represented part of their clear intercellular heterogeneity at DNA level. There are more and larger chromosomal heterogeneity features are hard to analyze and require higher resolution data.

We then performed scRNA-seq on 720 HeLa cells, hoping to build a featured landscape of transcriptome clusters. We identified 6 clusters and most highly expressed genes in these clusters functioned in different biology processes such as proliferation. Our results detected a rapidly proliferating cell population (Fig 3 cluster 2) and a population with interferon-induced characteristics (Fig 3 cluster 5). These results suggested the heterogeneity of transcriptome within HeLa cells could be due to different cellular properties and deserve further detailed investigation. We further utilized the scRNA-seq data to infer its copy number to see their correlation with the DNA data. We found a good correspondence between gene expression and copy number on chromosome 19, and the overall correlation between gene expression and copy number variation is also reasonable. It demonstrated that the CNV change at DNA level could reflect on the gene expression at RNA level.

In summary, our research has for the first time depicted the heterogeneity on both genome and transcriptome of HeLa-CCL2 cells at the single cell level. MALBAC results gave us an overall observation on intercellular genome heterogeneity. The transcriptome diversities showed featured clusters within HeLa-CCL2. The relationship between DNA and RNA sequencing results indicated that CNV could be reflected on gene expression (RNA-seq) levels. Although it is useful, we should also be cautious to perform and interpret single-cell sequencing experiments with HeLa or other very heterogeneous cell lines.

## Supporting information

S1 Fig The frequency of chromosome number. Mostly (85%) concentrated at 60–70.
(TIF)

S2 Fig MDA results of a HeLa-CCL2 cell. The same y axis and x axis with Fig 1, the results showed low resolution.
(TIF)

S1 Table Chromosome number of 150 cells in metaphase. A-E represented 5 karyotype stain sheets.
(XLSX)
