# Supplementary figures and images for: HeLa-CCL2 cell heterogeneity studied by single-cell DNA and RNA sequencing
Source: PLoS One. 2019 Dec 2;14(12):e0225466. doi: 10.1371/journal.pone.0225466 (PMC6886862; doi:10.1371/journal.pone.0225466)

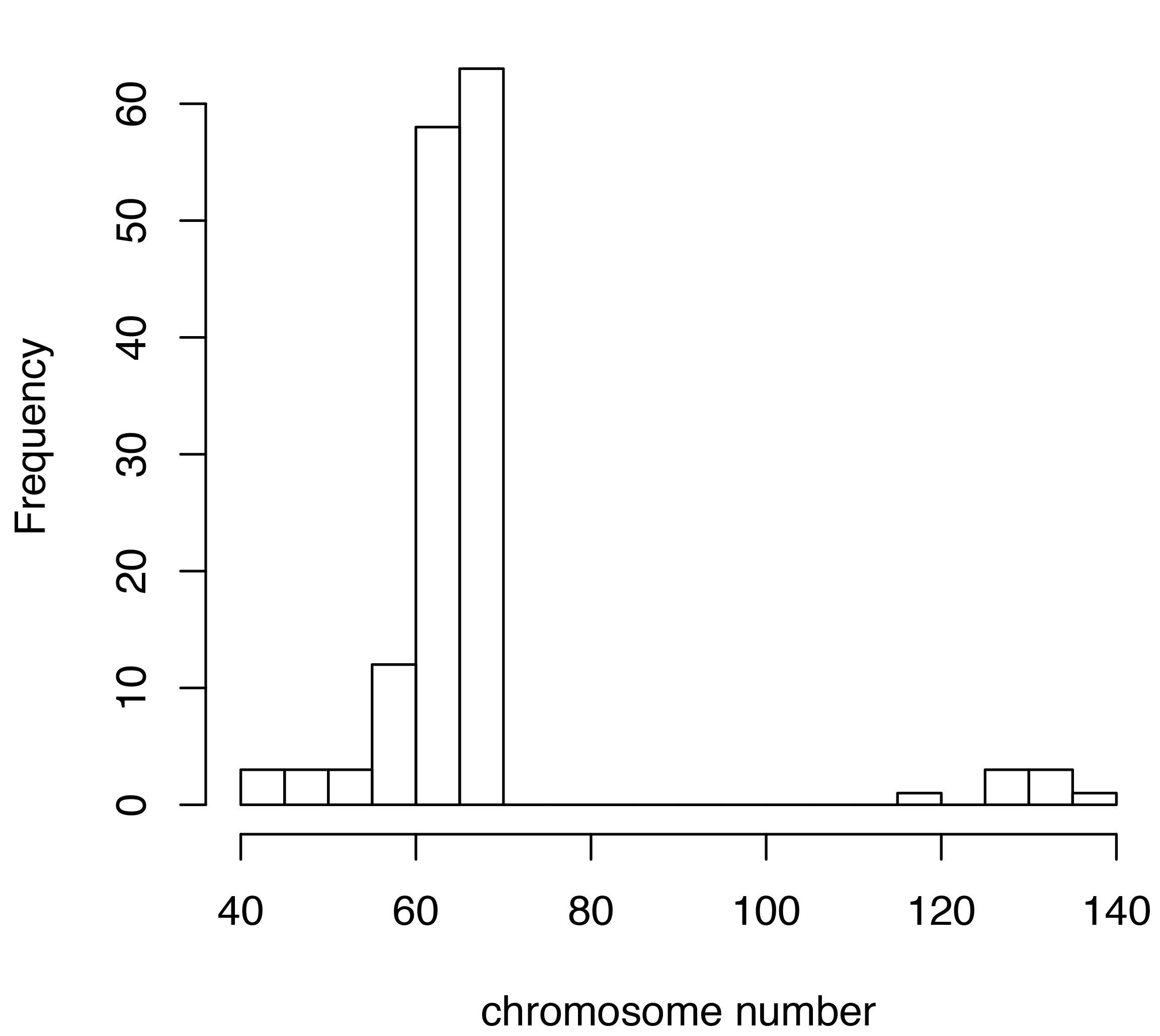

Supplement: S1 Fig — Mostly (85%) concentrated at 60–70. (TIF) [file pone.0225466.s001.tif]

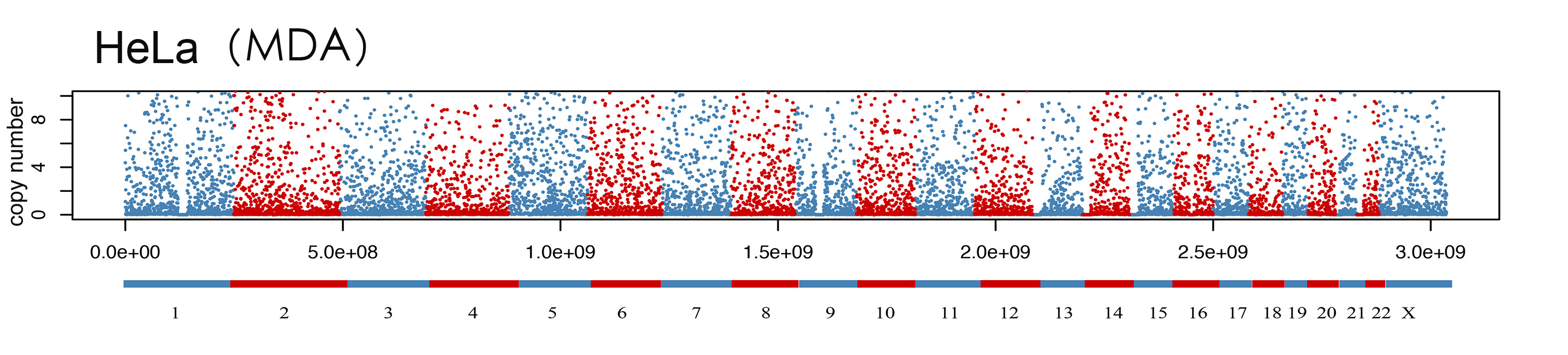

Supplement: S2 Fig — The same y axis and x axis with Fig 1, the results showed low resolution. (TIF) [file pone.0225466.s002.tif]
